# The Relationship between Knee Adduction Moment and Knee Osteoarthritis Symptoms according to Static Alignment and Pelvic Drop

**DOI:** 10.1155/2019/7603249

**Published:** 2019-12-26

**Authors:** Yong Nie, Hua Wang, Bin Xu, ZongKe Zhou, Bin Shen, FuXing Pei

**Affiliations:** ^1^Department of Orthopedic Surgery and National Clinical Research Center for Geriatrics, West China Hospital, West China Medical School, Sichuan University, Chengdu, Sichuan Province, China; ^2^The Third People's Hospital of Xindu District, Chengdu, Sichuan Province, China

## Abstract

**Objectives:**

To investigate the relationship between external knee adduction moment (KAM) and knee osteoarthritis (OA) symptoms according to static alignment and pelvic drop.

**Methods:**

Ninety-five participants with symptomatic knee OA were included. Radiographic severity was graded by Kellgren and Lawrence (KL) scale. The hip-knee-ankle (HKA) angle was used to assess limb alignment from a full-length lower-limb radiograph. KAM-related variables (peak KAM and KAM impulse) and pelvic drop angle were determined from 3D gait analysis. Symptoms were assessed via visual analog scale (VAS) for pain and hospital for special surgery (HSS) score for physical function. The relationship between KAM and symptoms was evaluated according to radiographic severity and pelvic drop using linear models.

**Results:**

According to the more affected knee in the varus group, both the two KAM-related measures (peak KAM and KAM impulse) were positively associated with greater VAS pain and were negatively associated with HSS score. Only peak KAM was correlated with VAS and HSS in the valgus group. VAS pain score of the more affected knee was positively correlated with pelvic drop angle. Stratified by pelvic drop angle, KAM-related variables were more positively associated with VAS pain and negatively associated with HSS score for patients with pelvic drop angle ≤3 degrees. The relationships between KAM and symptoms according to radiographic disease severity remained confusing.

**Conclusions:**

Static alignment and pelvic drop angle significantly affected relationships between KAM-related variables and knee OA symptoms, which may explain the confusing results as shown by previous studies.

## 1. Introduction

Knee osteoarthritis (OA) is a major cause of pain and physical disability [1]. The external knee adduction moment (KAM), which reflects medial-to-lateral knee joint load distribution during gait, has become an OA treatment target [2–4]. The KAM (peak and impulse) is a strong predictor of presence [5], severity [6–8], and the rate of progression [9] of knee OA [10]. Patients with medial compartment OA tend to have a higher peak KAM. This has led to a plethora of treatment options that attempt to lower the peak KAM [11]. Despite the use of the KAM as a biomechanical treatment target, the relationships between KAM-related variables and knee OA symptoms (pain and function) remain unclear [12].

Several studies showed positive correlations between the KAM and knee pain [13–16], while other investigations demonstrated inverse associations [17]. With respect to relationships between KAM and knee joint function, evidences were also conflicting [14, 15, 17]. Recent studies considered that relationships between KAM-related variables and symptoms may differ according to underlying structural knee OA severity, and tried to explain the inconsistent findings of studies to date [10, 12]. However, findings from the recent studies were also inconsistent. Henriksen et al. reported that patients with severity of knee OA ≤ KL grade 2 showed negative relationships between KAM and pain, while those with severity of knee OA > KL grade 2 showed a positive relationship between pain and KAM impulse [10]. However, Hall et al. reported that patients with knee OA of KL grade 2 showed no associations between KAM and knee pain or physical function, and those with knee OA of KL grade 4 demonstrated a negative relationship between KAM impulse and knee pain [12].

Failure to consider the determinative factors of KAM may account for the inconsistent findings from existing studies. First of all, the KAM during gait in subjects with knee OA is more closely correlated with static lower-limb alignment than radiographic disease severity [17]. The static alignment predicted 58% of the variance in the first peak KAM [11] and indicates that the weight of lower-limb alignment is highest in KAM calculation. In addition, patients with more severe knee OA have severe varus or valgus deformity. Thus, using radiographic disease severity to stratify the subjects may probably break the continuity of static alignment and cause a relatively narrow range of static alignment in each subgroup in statistical analyses. Therefore, in each subgroup, the narrowed range of static alignment may probably lead to bias. Furthermore, the specificity of KAM for patients with varus and valgus knee remains unclear [14]. The varus and valgus deformities were not distinguished in previous studies [10, 12, 13, 15, 16]. The correlations between KAM and symptoms in patients with varus and valgus knee may probably be different.

Second of all, the ground reaction force predicted the second most variance in the first peak KAM [11]. The pelvic drop or compensatory trunk movements as a result of joint pain directly affect the direction and magnitude of ground reaction force and thus affect KAM of both sides [18, 19]. However, to date, no research has stratified the patients by static alignments and the pelvic drop simultaneously to investigate the relationships between KAM-related variables and knee OA symptoms. This information is important for understanding the clinical indications of using KAM to target treatments for patients with knee OA.

The purpose of this study was to investigate the associations between KAM-related variables (peak KAM and KAM impulse) and knee OA symptoms (pain and physical function score) according to static alignment and pelvic drop.

## 2. Methods

### 2.1. Patients

Ninety-five patients with knee OA were included. Participants were recruited from July 2014 to October 2016 via community advertisements. Inclusion criteria included: (1) predominance of self-reported pain on most days of the month; (2) definite bilateral radiographic tibiofemoral joint OA defined as KL grade ≥ 2 [20]; and (3) medial knee OA with varus deformity or lateral knee OA with valgus deformity for the more affected side.

Exclusion criteria included: (1) history of intra-articular corticosteroid injection or knee surgery; (2) systemic arthritic condition (rheumatoid arthritis, etc); (3) any other muscular, joint, or neurological condition influencing lower limb; (4) biomechanical conservative treatments (lateral wedge insoles, knee brace, gait modification with toe out or toe in, etc); (5) unable to walk without aid; (6) body mass index (BMI) ≥ 30 kg/m^2^; and (7) spine/pelvis/hip/ankle/foot pain/pathology. The Human Research Ethics Committee approved the study, and all participants provided written informed consent.

### 2.2. Radiographs

Biplane (anteroposterior and lateral) weight-bearing semiflexed (15°) knee joint radiographs were obtained. The radiographic disease severity of the tibiofemoral OA was assessed with the KL system [20] by an expert orthopedic surgeon. In the KL grading system, disease severity is rated on a five-point scale from grade 0 (no sign of OA) to grade 4 (severe OA). In our present study, patients were eligible if they were graded as either “KL2” (definitive osteophytes with possible narrowing of joint space), “KL3” (moderate multiple osteophytes, definite narrowing of joint space and some sclerosis and possible deformity of bone ends), or “KL4” (large osteophytes, marked narrowing of joint space, severe sclerosis, and definite deformity of bone ends) [20].

The hip-knee-ankle (HKA) angle was used to assess static limb alignment from a full-length lower-limb radiograph during standing [21, 22]. The HKA angle was defined as the angle between the mechanical axes of the femur (from the center of the femoral head to the midpoint of the tibial plateau) and the tibia (from the midpoint of the tibial plateau to the midpoint of the ankle) and measured in the frontal plane [21, 22]. Varus and valgus malalignment were noted by HKA values >0° and <0°, respectively; neutral alignment was denoted by 0° [17].

### 2.3. Pain and Physical Function

Knee pain during walking over the previous weeks was measured by visual analog scale (VAS) from 0 (no pain) to 10 (worst imaginable pain) in 1 cm intervals for both sides [23]. The hospital for special surgery (HSS) knee score was used to investigate functional status [24]. The widely used HSS emphasizes pain, function, and range of motion and is known for its high interobserver correlation [25]. The HSS scoring system includes the following subscores: pain (30 points), function (32 points—walking, stair climbing, transfer activity, and muscle strength), and knee (38 points—range of motion, instability, and flexion deformity). Subtractions are made for the use of crutches, extension lags, and misalignment >5°. Both the VAS and HSS scoring systems have been reported as reliable in patients with knee OA [23, 25]. In addition, passive flexion/extension range of motion (RoM) of the knee joint was measured using a standard clinical goniometer with the patient lying supine.

### 2.4. Gait Analyses, KAM Variables, and Pelvic Drop Angle

All gait trials were completed with shoes off. An initial standing static trial was performed using 28 retroreflective markers on the pelvis, each thigh, lower leg, and foot and additional 10 markers placed over the ankle, femoral epicondyles, and greater trochanter to determine segment orientations. Patients then walked at a self-selected waking speed on a 12 m walkway wearing 28 markers to track the motions of the pelvis, each thigh, lower leg, and foot [26]. Three-dimensional trajectories of the markers were collected at 290 Hz using a 10-camera motion analysis system (Oqus300, Qualisys, Gothenburg, Sweden). Ground reaction forces (GRFs) were recorded using two force plates (Bertec, Columbus, OH, USA) incorporated into the walkway. Each patient performed three gait trials (with enough interval time for each patient to avoid the effects of fatigue or symptom exacerbation from multiple repetitions) and was instructed to walk as naturally as possible looking straight ahead [27]. After three trials, we investigated if each was within ±5% of the average speed of the three trials. We stopped collecting additional data when three continuous trials within ±5% average walking speed were obtained.

Using inverse dynamic techniques and commercially available software (Visual 3D, C-Motion, Inc., Rockville, MD), external knee adduction moment (KAM) normalized to body weight (BW) and height (HT) was calculated. The KAM impulse for each gait trial was calculated by integrating the stance phase portion of the KAM waveform using a custom-written software program MATLAB (version 7.1, MathWorks, Inc., MA, USA) according to the following equation:

(1) $$\begin{array}{c} \text{KAM impulse}=\int_{a}^{b}\text{KAM}\left(t\right)\text{d}t, \end{array}$$

where KAM (*t*) represents KAM at time (*t*), *a* denotes time (*t*) at heel strike, and *b* is time (*t*) at toe off [28].

The peak KAM (Nm/(BW × HT)%) and the positive KAM angular impulse (Nm·s/(BW × HT)%) were averaged over three trials.

The pelvic drop angle was defined as the maximum angle in the frontal plane during the single-limb stance time of the more affected side [18]. According to the maximum pelvic drop angle (3 degrees) during gait of healthy controls [18], patients were stratified into two grades: pelvic drop angle ≤3 degrees and >3 degrees.

### 2.5. Statistical Analysis

Patients were divided into two groups: varus group (HKA > 0°) and valgus group (HKA < 0°) [17], according to the HKA angle of the more affected side. Paired *t*-tests were used to investigate the differences between two sides in clinical and biomechanical variables. Linear regression was performed to examine the associations between KAM-related variables and symptoms (pain and physical function score) for the varus and valgus groups and the relationship between pelvic drop angle and VAS pain. One-way analysis of variance (for continuous variables) and Pearson chi-squared (for categorical variables) tests were used to compare demographic variables, clinical measures, and biomechanical measures across the three grades of knee OA severity and two levels of pelvic drop. Using linear models, relationships between KAM-related variables (independent variables) and measures of pain and physical function (dependent variables) were evaluated according to three grades of knee OA severity and two levels of pelvic drop. Regression models were unadjusted and adjusted for age and walking speed [10, 12]. SPSS version 19 was used for statistical analysis and significance was set at *P* < 0.05.

## 3. Results

Seventy-seven and eighteen patients with knee OA were in the varus and valgus group, respectively. Patients' characteristics are listed in Table 1. In general, the cohort was middle-aged, normal weight, and almost represented female. The more affected side showed more severity of knee OA, more pain, more severe varus or valgus deformity, and less passive RoM than the contralateral side. The more affected side also had larger KAM than the contralateral side in the varus group.

According to the more affected side in the varus group, both the two KAM-related measures (peak KAM and KAM impulse) were positively associated with greater VAS pain (*r* = 0.293, *P* < 0.001; *r* = 0.380, *P* < 0.001, respectively) and were negatively associated with HSS score (*r* = −0.149, *P*=0.045; *r* = −0.253, *P*=0.002, respectively). In addition, the lower peak KAM was associated with greater VAS pain (*r* = −0.446, *P*=0.006) and was associated with lower HSS score (*r* = 0.340, *P*=0.043) for the more affected side in the valgus group (Table 2).

**Figure 1:**
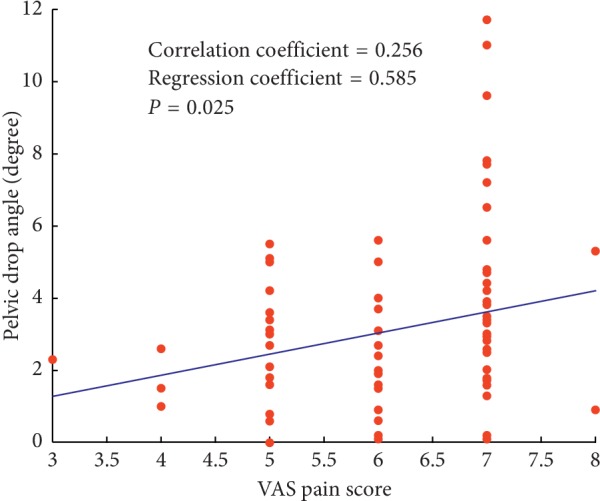
Linear relationships between maximum pelvic drop angle during gait and VAS pain score in the varus group.

In the varus group, VAS pain score of the more affected knee was positively associated with pelvic drop angle (*r* = 0.256, regression coefficient = 0.585, *P*=0.025) (Figure 1).  Table 3 presents descriptive characteristics for the varus group according to radiographic disease severity and pelvic drop angle. With the KL grade stratified strategy, all the clinical and biomechanical variables were also stratified into three levels. However, the HKA angle, peak KAM, and KAM impulse were on a similar level across grades of pelvic drop angle. Below we present unadjusted and covariate-adjusted parameter estimates for each level of radiographic disease severity and pelvic drop angle.

### 3.1. KAM versus VAS Pain

For the unadjusted and two covariate-adjusted models, there were significant positive associations observed between KAM-related variables and VAS pain for patients with pelvic drop angle ≤3 degrees (*P*=0.001, *P*=0.001, and *P*=0.002, respectively, for peak KAM; *P* < 0.001, *P*=0.001, and *P*=0.009, respectively, for KAM impulse) (Table 4). However, there were no statistically significant associations observed between any KAM-related variables and VAS pain for patients with three KL grade OA disease, except the adjusted model with age and speed for KL grade 2 (*P* < 0.001), in which increased KAM-related variables were significantly associated with lower VAS pain (Table 4).

### 3.2. KAM versus HSS Score

For all three models, there were significant negative correlations observed between KAM-related variables and HSS score for patients with pelvic drop angle ≤3 degrees (*P*=0.008, *P*=0.028, and *P*=0.048, respectively, for peak KAM; *P*=0.001, *P*=0.006, and *P*=0.011, respectively, for KAM impulse) (Table 5). Although there were statistically negative associations observed between KAM-related variables and HSS score for patients with KL grade 2 (*P*=0.006, *P* < 0.001, and *P* < 0.001, respectively, for peak KAM; *P*=0.061, *P*=0.001, and *P* < 0.001, respectively, for KAM impulse), no statistically significant correlation was observed between any KAM-related parameters and HSS score for patients with KL grades 3 and 4 (Table 5).

## 4. Discussion

Evidences of relationships between KAM and symptoms (VAS pain and HSS score for physical function) were conflicting. Failure to consider the determinative factors of KAM may account for the inconsistent findings from existing studies. KAM is determined by GRF and lever arm. The lever arm is determined by lower-limb alignment and the position of center of gravity (affected by compensatory pelvic or trunk movements) [29]. Schmitz and Noehren demonstrated that the knee adduction angle predicted 58% of the variance in the first peak KAM and the GRF predicted the second most variance [11]. Therefore, lower-limb alignment and GRF-related factors should be stratified to precisely investigate the clinical associations between KAM-related variables and knee OA symptoms.

Previous studies investigating the associations between KAM and symptoms (VAS pain and HSS score for physical function) did not distinguish varus and valgus knees from subjects [10, 12, 13, 15–17]. The clinical sensitivity of KAM for patients with varus and valgus knee remains unclear [14]. Therefore, previous studies may cause bias in their conclusions. In our study, 95 patients were divided into two groups: varus group (HKA > 0°) and valgus group (HKA < 0°), according to the HKA angle of the more affected side [17]. In the varus group, our findings indicated that both the two KAM-related variables (peak KAM and KAM impulse) of the more affected side were positively associated with knee pain, while negatively associated with HSS score. However, in the valgus group, only the peak KAM was correlated with knee pain and HSS score. There was no significant correlation between KAM impulse and knee pain or HSS score in the valgus group. Therefore, the clinical sensitivity of KAM is probably higher for patients with varus knee than valgus knee.

With respect to GRF-related factors, the adoption of compensatory gait (pelvic drop and compensatory trunk movements) because of knee pain by subjects with knee OA changes the position of center of gravity, thus affects the lever arm of GRF, and leads to changes of KAM [14]. Stief et al. reported that ipsilateral trunk lean caused decreased KAM because of GRF vector shifting to knee joint center [19]. In addition, Dunphy et al. demonstrated that contralateral pelvic drop during gait affected KAM [18]. Since the compensatory mechanisms are adopted by patients with knee OA to reduce KAM on the more affected side, the KAM values measured in gait analysis may not reflect the severity of symptoms (VAS pain and HSS score for physical function) attributable to OA [14]. Furthermore, in our study, we also demonstrated that higher VAS pain score of the severe knee was significantly associated with a larger pelvic drop angle. According to the maximum pelvic drop angle (3 degrees) during walking from healthy controls [18], patients with maximum pelvic drop angle ≤3 degrees during walking do not adopt compensatory gait and have stable position of center of gravity, thus do not affect the lever arm of GRF, and do not lead to changes of KAM. However, for patients with maximum pelvic drop angle >3 degrees during walking, compensatory gait will lead to changes of KAM. Therefore, in our study, patients with varus knee were stratified into two grades: pelvic drop angle ≤3 degrees and >3 degrees, respectively, to better evaluate whether pelvic drop affects associations between KAM-related variables (peak KAM and KAM impulse) and knee OA symptoms (pain and physical function score). In addition, we also investigated these associations according to radiographic disease severity for comparison.

Our findings demonstrated that pelvic drop angle significantly affected relationships between KAM-related variables (peak KAM and KAM impulse) and knee OA symptoms (pain and physical function score). With the stratified strategy by pelvic drop angle, our findings indicated that the range of HKA angle was larger than that when stratified by KL grade and thus assured the continuity and integrity of HKA angle data in statistical analysis. Furthermore, the HKA angle, peak KAM, and KAM impulse were on a similar level across grades of pelvic drop angle. KAM-related variables were positively associated with VAS pain and negatively associated with physical function (HSS score) for patients with maximum pelvic drop angle ≤3 degrees, which were not found in condition of maximum pelvic drop angle >3 degrees. Patients with maximum pelvic drop angle >3 degrees (including contralateral and ipsilateral pelvic drop) during compensatory gait had unnormal position of center of gravity and led to unnormal changes to KAM and thus disturbed the relationship between KAM and pain or physical function score. When the patients with pelvic drop angle >3 degrees were excluded, the correlation coefficients between KAM-related variables and pain or physical function were higher than that found in all patients. Therefore, the maximum pelvic drop angle (less than 3 degrees) obtained from the gait of healthy controls [18] could be used as an indicator for using KAM to target treatments for patients with varus knee OA to gain symptomatic benefits.

Compared with the explicit results of the stratified strategy of pelvic drop angle, the relationships between KAM-related variables and symptoms according to radiographic disease severity remained confusing as shown by our and previous studies [10, 12]. With the KL grade stratified strategy, our study showed that all the clinical and biomechanical variables were also stratified into three levels. This strategy caused different mean values and ranges for these variables in each level and broke the continuity of these variables in statistical analyses. This may explain the confusing results of KL grade stratified strategy. However, it was plausible that, for knee OA patients with KL grade 2, the KAM-related variables were negatively associated with pain and physical function, which is in agreement with the previous study [10].

There are also limitations of our study. First, the trunk movements were not measured in 3D gait analyses. Future study including trunk movement would further contribute to evaluate the effects of trunk movement on the relationships between KAM-related variables and symptoms. Second, the medial knee contact force was not measured or calculated. Although the KAM is widely used to infer medial knee joint loading, our findings only indicated the relationships between KAM-related variables and symptoms. Third, the sample size and the range of alignment (HKA angle) for knee OA patients with valgus knee were relatively small and narrow, respectively. Thus, our study did not further investigate the clinical relationships between variables in these patients. More knee OA patients with valgus knee will be included in our further studies.

In conclusion, our study showed that static alignment and pelvic drop angle significantly affected relationships between KAM-related variables and knee OA symptoms, which may explain the confusing results as shown by previous studies. We also found evidence to contribute to further understand the clinical indications of using KAM to target treatments for patients with knee OA to gain symptomatic benefits. The indications may probably point to medial knee OA patients with varus deformity and normal maximum pelvic drop angle (less than 3 degrees) during walking.

## Figures and Tables

**Table 1:** Patients' characteristics.

Characteristics	Total group *n* = 95			
	Varus (*n* = 77)		Valgus (*n* = 18)	
	More affected side	Contralateral side	More affected side	Contralateral side
Demographic variables				
Age (years)	63.1 (10.3)		59.2 (11.3)	
Women, *n* (%)	68 (88.3%)		17 (94.4%)	
Height (m)	1.57 (0.06)		1.58 (0.05)	
Weight (kg)	61.3 (10.2)		61.6 (12.4)	
BMI (kg/m^2^)	24.8 (3.7)		24.6 (4.1)	
Clinical variables				
KL 2/3/4 (*n*)	14/26/37	15/39/23	0/10/8	3/11/4
KL grade	3.6 (0.6)^*∗*^	3.2 (0.6)	3.4 (0.5)^*∗*^	3.1 (0.6)
Alignment (HKA angle) (degrees)	5.9 (4.6)^*∗*^	3.7 (3.8)	−2.5 (2.9)^*∗*^	0.1 (2.1)
VAS pain (0–10)	6.1 (1.0)^*∗*^	2.5 (2.0)	5.4 (1.2)^*∗*^	2.2 (1.8)
HSS score (0–100)	56.9 (10.4)		57.9 (10.5)	
Passive flexion/extension RoM (degrees)	107.0 (17.0)^*∗*^	113.4 (14.4)	106.9 (25.8)	113.9 (15.9)
Biomechanical variables				
Self-selected walking speed (m/s)	0.70 (0.22)		0.83 (0.21)	
Peak KAM (Nm/(BW × HT)%)	3.45 (1.14)^*∗*^	3.08 (1.12)	2.25 (0.93)	2.34 (1.11)
KAM impulse (Nm·s/(BW × HT)%)	1.79 (1.00)^*∗*^	1.58 (0.96)	0.88 (0.55)	1.03 (0.61)

KL: Kellgren and Lawrence; HKA: hip-knee-ankle; VAS: visual analog scale; HSS: the hospital for special surgery; RoM: range of motion; KAM: knee adduction moment; BW: body weight; HT: height. ^*∗*^Significantly different to the contralateral side (*P* < 0.05). Means (standard deviations) are provided above for variables.

**Table 2:** Linear relationships between KAM-related measures (independent variable) and VAS pain/HSS score (dependent variable) according to the more affected side in the varus and valgus groups.

KAM-related measures	VAS pain		HSS score	
	Correlation coefficient	Slope *P* value	Correlation coefficient	Slope *P* value
Peak KAM (Nm/(BW × HT)%)				
Varus group	0.293	**≤0.001**	−0.149	**0.045**
Valgus group	−0.446	**0.006**	0.340	**0.043**
KAM impulse (Nm·s/(BW × HT)%)				
Varus group	0.380	**≤0.001**	−0.253	**0.002**
Valgus group	−0.277	0.102	0.112	0.516

KAM: knee adduction moment; BW: body weight; HT: height; VAS: visual analog scale; HSS: the hospital for special surgery.

**Table 3:** Characteristics of patients stratified by KL grade and pelvic drop angle according to the more affected side in the varus group.

Characteristics	Stratified by KL grade			Stratified by pelvic drop angle (PDA)	
	KL grade 2 *n* = 14	KL grade 3 *n* = 26	KL grade 4 *n* = 37	PDA ≤ 3° *n* = 45	PDA > 3° *n* = 32
Demographic variables					
Age (years)	48.3 (7.3)	57.0 (9.9)‖	67.8 (7.5)¶	61.6 (11.6)	65.3 (7.8)§
Women, *n* (%)	11 (78.6%)	25 (96.2%)	32 (86.5%)	38 (84.4%)	30 (93.8%)
Height (m)	1.59 (0.05)	1.57 (0.04)	1.57 (0.07)	1.58 (0.06)	1.55 (0.06)§
Weight (kg)	59.6 (5.1)	61.9 (7.6)	61.1 (11.7)	61.4 (11.0)	61.1 (9.1)
BMI (kg/m^2^)	23.6 (1.4)	25.2 (3.2)	24.8 (4.0)	24.4 (3.5)	25.5 (3.8)
Clinical variables					
Alignment (HKA angle) (degrees)	1.9 (1.5)	2.6 (2.1)	8.1 (4.4)¶	5.4 (4.2)	6.7 (5.0)
VAS pain (0–10)	5.0 (0.8)	5.3 (0.9)	6.7 (0.7)¶	6.0 (1.1)	6.3 (0.9)§
HSS score (0–100)	69.3 (5.3)	64.7 (7.3)	51.6 (8.5)¶	58.1 (9.7)	55.3 (11.2)§
Passive RoM (degrees)	120.0 (13.1)	120.4 (10.6)	98.5 (14.6)¶	111.6 (15.2)	100.6 (17.5)§
Biomechanical variables					
Self-selected walking speed (m/s)	0.98 (0.18)	0.78 (0.21)‖	0.63 (0.19)¶	0.73 (0.21)	0.65 (0.22)§
Peak KAM (Nm/(BW × HT)%)	3.45 (0.71)	2.90 (1.10)	3.75 (1.08)¶	3.39 (1.18)	3.53 (1.09)
KAM impulse (Nm·s/(BW × HT)%)	1.29 (0.48)	1.27 (0.69)	2.12 (1.04)¶	1.66 (0.91)	1.97 (1.11)

KL: Kellgren and Lawrence; PDA: pelvic drop angle; HKA: hip-knee-ankle; VAS: visual analog scale; HSS: the hospital for special surgery; RoM: range of motion; KAM: knee adduction moment; BW: body weight; HT: height; “‖” means significantly different to KL grade 2 (*P* < 0.05); “¶” means significantly different to KL grade 3 (*P* < 0.05); “§” means significantly different to PDA ≤ 3° (*P* < 0.05). Means (standard deviations) are provided above for variables.

**Table 4:** Linear relationships between KAM-related measures (independent variable) and VAS pain (dependent variable) according to radiographic disease severity and pelvic drop angle (PDA) in the varus group.

VAS pain	Univariable analysis			Multivariable analysis^*∗*^			Multivariable analysis^†^		
	Correlation coefficient	Regression coefficient	Slope *P* value	Correlation coefficient	Regression coefficient	Slope *P* value	Correlation coefficient	Regression coefficient	Slope *P* value
Peak KAM (Nm/(BW × HT)%)									
KL grade 2 (*n* = 14)	−0.102	−0.109	0.811	−0.681	−0.729	0.104	−0.769	−0.823	**≤0.001**
KL grade 3 (*n* = 26)	0.019	0.015	0.894	0.160	0.125	0.153	0.183	0.144	0.109
KL grade 4 (*n* = 37)	0.175	0.111	0.091	0.179	0.113	0.082	0.163	0.103	0.116
PDA ≤ 3 (*n* = 45)	0.346	0.326	**0.001**	0.284	0.268	**0.001**	0.262	0.247	**0.002**
PDA > 3 (*n* = 32)	0.175	0.144	0.167	0.127	0.104	0.205	0.151	0.125	0.126
KAM impulse (Nm·s/(BW × HT)%)									
KL grade 2 (*n* = 14)	0.158	0.250	0.709	−0.813	−1.284	0.160	−0.878	−1.645	**≤0.001**
KL grade 3 (*n* = 26)	0.096	0.119	0.500	0.144	0.180	0.189	0.133	0.166	0.248
KL grade 4 (*n* = 37)	0.172	0.113	0.098	0.169	0.111	0.102	0.132	0.087	0.274
PDA ≤ 3 (*n* = 45)	0.388	0.475	**≤0.001**	0.275	0.336	**0.001**	0.255	0.312	**0.009**
PDA > 3 (*n* = 32)	0.347	0.280	**0.005**	0.190	0.153	0.066	0.139	0.112	0.207

KL: Kellgren and Lawrence; PDA: pelvic drop angle; KAM: knee adduction moment; BW: body weight; HT: height; VAS: visual analog scale. ^*∗*^Age; ^†^age, speed.

**Table 5:** Linear relationships between KAM-related measures (independent variable) and HSS score (dependent variable) according to radiographic disease severity and pelvic drop angle (PDA) in the varus group.

HSS score	Univariable analysis			Multivariable analysis^*∗*^			Multivariable analysis^†^		
	Correlation coefficient	Regression coefficient	Slope *P* value	Correlation coefficient	Regression coefficient	Slope *P* value	Correlation coefficient	Regression coefficient	Slope *P* value
Peak KAM (Nm/(BW × HT)%)									
KL grade 2 (*n* = 14)	−0.864	−6.500	**0.006**	−0.875	-9.101	**≤0.001**	−0.899	−9.306	**≤0.001**
KL grade 3 (*n* = 26)	0.150	0.991	0.288	0.147	0.973	0.313	0.084	0.555	0.556
KL grade 4 (*n* = 37)	0.069	0.541	0.510	0.065	0.511	0.530	0.070	0.549	0.505
PDA ≤ 3 (*n* = 45)	−0.209	−1.725	**0.008**	−0.163	−1.345	**0.028**	−0.142	−1.174	**0.048**
PDA > 3 (*n* = 32)	−0.055	−0.566	0.667	−0.019	−0.199	0.867	−0.047	−0.481	0.681
KAM impulse (Nm·s/(BW × HT)%)									
KL grade 2 (*n* = 14)	−0.684	−7.597	0.061	−0.785	−17.530	**0.001**	−0.863	−18.594	**≤0.001**
KL grade 3 (*n* = 26)	−0.110	−1.157	0.437	−0.114	−1.198	0.427	−0.026	−0.275	0.855
KL grade 4 (*n* = 37)	0.076	0.621	0.466	0.080	0.650	0.441	0.129	1.053	0.290
PDA ≤ 3 (*n* = 45)	−0.265	−2.847	**0.001**	−0.181	−1.944	**0.006**	−0.148	−1.591	**0.011**
PDA > 3 (*n* = 32)	−0.210	−2.118	0.096	−0.092	−0.925	0.441	−0.008	−0.077	0.952

KL: Kellgren and Lawrence; PDA: pelvic drop angle; KAM: knee adduction moment; BW: body weight; HT: height; HSS: the hospital for special surgery. ^*∗*^Age; ^†^age, speed.

## Data Availability

Original data supporting the results are available from the corresponding author upon request if needed.

## Additional Points

*Article Focus*. KAM as a knee OA treatment target may have indications; the relationships between KAM-related variables and knee OA symptoms (pain and function) according to KL grades remain confusing; static alignment and pelvic drop play an important role in determining KAM. *Key Messages*. Static alignment and pelvic drop significantly affect relationships between KAM and knee OA symptoms; varus knees and pelvic drop ≤3° should be considered using KAM as the knee OA treatment target; the relationships between KAM and knee OA symptoms become confusing according to stratified OA severity. *Strengths and Limitations*. Sample size for knee OA patients with varus knee was large; solid evidences show the different relationships between KAM and knee OA symptoms according to stratified static alignment and pelvic drop; the trunk movements were not measured in 3D gait analyses; the sample size for knee OA patients with valgus knee was relatively small.

## References

[1] Hunter D. J., McDougall J. J., Keefe F. J. (2008). The symptoms of osteoarthritis and the genesis of pain. *Rheumatic Disease Clinics of North America*.

[2] Hinman R. S., Wrigley T. V., Metcalf B. R. (2014). Unloading shoes for osteoarthritis of the knee: protocol for the SHARK randomised controlled trial. *BMC Musculoskeletal Disorders*.

[3] Moyer R. F., Birmingham T. B., Bryant D. M., Giffin J. R., Marriott K. A., Leitch K. M. (2015). Biomechanical effects of valgus knee bracing: a systematic review and meta-analysis. *Osteoarthritis and Cartilage*.

[4] Bennell K. L., Kyriakides M., Metcalf B. (2014). Neuromuscular versus quadriceps strengthening exercise in patients with medial knee osteoarthritis and varus malalignment: a randomized controlled trial. *Arthritis & Rheumatology*.

[5] Baliunas A. J., Hurwitz D. E., Ryals A. B. (2002). Increased knee joint loads during walking are present in subjects with knee osteoarthritis. *Osteoarthritis and Cartilage*.

[6] Henriksen M., Graven-Nielsen T., Aaboe J., Andriacchi T. P., Bliddal H. (2010). Gait changes in patients with knee osteoarthritis are replicated by experimental knee pain. *Arthritis Care & Research*.

[7] Mündermann A., Dyrby C. O., Andriacchi T. P. (2005). Secondary gait changes in patients with medial compartment knee osteoarthritis: increased load at the ankle, knee, and hip during walking. *Arthritis & Rheumatism*.

[8] Mündermann A., Dyrby C. O., Hurwitz D. E., Sharma L., Andriacchi T. P. (2004). Potential strategies to reduce medial compartment loading in patients with knee osteoarthritis of varying severity: reduced walking speed. *Arthritis & Rheumatism*.

[9] Miyazaki T., Wada M., Kawahara H., Sato M., Baba H., Shimada S. (2002). Dynamic load at baseline can predict radiographic disease progression in medial compartment knee osteoarthritis. *Annals of the Rheumatic Diseases*.

[10] Henriksen M., Aaboe J., Bliddal H. (2012). The relationship between pain and dynamic knee joint loading in knee osteoarthritis varies with radiographic disease severity. A cross sectional study. *The Knee*.

[11] Schmitz A., Noehren B. (2014). What predicts the first peak of the knee adduction moment?. *The Knee*.

[12] Hall M., Bennell K. L., Wrigley T. V. (2017). The knee adduction moment and knee osteoarthritis symptoms: relationships according to radiographic disease severity. *Osteoarthritis and Cartilage*.

[13] Amin S., Luepongsak N., McGibbon C. A., LaValley M. P., Krebs D. E., Felson D. T. (2004). Knee adduction moment and development of chronic knee pain in elders. *Arthritis Care & Research*.

[14] Kim W. Y., Richards J., Jones R. K., Hegab A. (2004). A new biomechanical model for the functional assessment of knee osteoarthritis. *The Knee*.

[15] Kito N., Shinkoda K., Yamasaki T. (2010). Contribution of knee adduction moment impulse to pain and disability in Japanese women with medial knee osteoarthritis. *Clinical Biomechanics*.

[16] Robbins S. M., Birmingham T. B., Callaghan J. P., Jones G. R., Chesworth B. M., Maly M. R. (2011). Association of pain with frequency and magnitude of knee loading in knee osteoarthritis. *Arthritis Care & Research*.

[17] Hurwitz D. E., Ryals A. B., Case J. P., Block J. A., Andriacchi T. P. (2002). The knee adduction moment during gait in subjects with knee osteoarthritis is more closely correlated with static alignment than radiographic disease severity, toe out angle and pain. *Journal of Orthopaedic Research*.

[18] Dunphy C., Casey S., Lomond A., Rutherford D. (2016). Contralateral pelvic drop during gait increases knee adduction moments of asymptomatic individuals. *Human Movement Science*.

[19] Stief F., Böhm H., Ebert C., Döderlein L., Meurer A. (2014). Effect of compensatory trunk movements on knee and hip joint loading during gait in children with different orthopedic pathologies. *Gait & Posture*.

[20] Kellgren J. H., Lawrence J. S. (1957). Radiological assessment of osteo-arthrosis. *Annals of the Rheumatic Diseases*.

[21] Cooke D., Scudamore A., Li J., Wyss U., Bryant T., Costigan P. (1997). Axial lower-limb alignment: comparison of knee geometry in normal volunteers and osteoarthritis patients. *Osteoarthritis and Cartilage*.

[22] Moreland J. R., Bassett L. W., Hanker G. J. (1987). Radiographic analysis of the axial alignment of the lower extremity. *The Journal of Bone & Joint Surgery*.

[23] Bellamy N. (1997). Osteoarthritis clinical trials: candidate variables and clinimetric properties. *The Journal of Rheumatology*.

[24] Davies A. P. (2002). Rating systems for total knee replacement. *The Knee*.

[25] Bach C. M., Nogler M., Steingruber I. E. (2002). Scoring systems in total knee arthroplasty. *Clinical Orthopaedics and Related Research*.

[26] Cappozzo A., Catani F., Della Croce U., Leardini A. (1995). Position and orientation in space of bones during movement: anatomical frame definition and determination. *Clinical Biomechanics*.

[27] Ewen A. M., Stewart S., St Clair Gibson A., Kashyap S. N., Caplan N. (2012). Post-operative gait analysis in total hip replacement patients-a review of current literature and meta-analysis. *Gait & Posture*.

[28] Thorp L. E., Sumner D. R., Block J. A., Moisio K. C., Shott S., Wimmer M. A. (2006). Knee joint loading differs in individuals with mild compared with moderate medial knee osteoarthritis. *Arthritis & Rheumatism*.

[29] Lewinson R. T., Worobets J. T., Stefanyshyn D. J. (2015). Calculation of external knee adduction moments: a comparison of an inverse dynamics approach and a simplified lever-arm approach. *The Knee*.

